# Avondale Disaster

**Published:** 1869-11

**Authors:** 


### AVONDALE DISASTER.

Much has been said respecting the sad accident at Avondale, by which 108 fellow-creatures lost their lives, simply for the want of proper precaution; and we cannot permit the subject to pass without calling attention to, at least, one practical method by which these brave men may have a chance for life.

At such places in the mine as may be deemed expedient, construct strong air-tight rooms (say to admit fifty persons each), with doors to close so as to exclude all air or gas from the mine.

Connect these air-tight rooms, by means of iron pipes, with an air-pump at the surface, having one pipe through which to supply fresh air to the room below, and a second pipe through which the foul air may escape. By an arrangement something after this plan, in case of accident, the miners might retreat to these rooms and there be safe until assistance could be rendered from above. Through these air-tight pipes messages might be conveyed to and from the mine, provisions and water supplied without the least difficulty, and life prolonged indefinitely.

We add our voice to that of the public generally, that Congress shall take immediate steps, on re-assembling, to enact such laws as shall compel mining companies to do something for the better protection of these men who risk their lives for our comfort.

As we go to press, still another frightful accident is chronicled in one of our western exchanges, where three men are precipitated down the shaft of a mine some 180 feet and mutilated in the most shocking manner by the breaking of a wire rope.

We suggest, as a simple and effectual means of preventing accidents of this sort, and used especially when human beings are freight, that a second rope, not bearing weight, but allowed to run loosely between the drum and the car carrying the men, be employed, so that whatever may occur to the main or hoisting-rope, this second or reserve rope shall be ready to take the weight and save life.

It is impossible to detect weakness in a *wire rope;* and though a rope, as in the foregoing instance, may have been used to sustain many thousand pounds during the entire day, and break with the weight of only a few hundred pounds, the first intimation would be its parting, caused by the granulation of the metal with constant bending.

